# Intracranial Structural Malformations in Children in Tibet: CT and MRI Findings in a Single Tertiary Center

**DOI:** 10.2174/0115734056321642241213103658

**Published:** 2025-01-02

**Authors:** Xuan Yin, Dawa Ciren, Ciren Guojie, Guofu Zhang, Jimei Wang, He Zhang

**Affiliations:** 1 Department of Radiology, Obstetrics and Gynecology Hospital, Fudan University, Shanghai, P.R.China; 2 Department of Radiology, Shigatse People's Hospital, Shigatse, Xianzang Autonomous Region, P.R.China; 3 Department of Neonatology, Obstetrics and Gynecology Hospital, Fudan University, Shanghai, P.R.China

**Keywords:** Infant, Newborn, Central nervous system, Abnormalities, Computed tomography, Magnetic resonance imaging

## Abstract

**Objectives:**

The objective of this study was to summarize the findings of children’s intracranial congenital or developmental malformations found during imaging procedures in the Tibetan plateau.

**Methods:**

We retrospectively reviewed the imaging data of the suspected patients who presented with the central nervous system (CNS) malformations and were enrolled either through the clinic or after ultrasound examinations between June 2019 and June 2023 in our institution. All imaging data were interpreted by two experienced radiologists through consensus reading.

**Results:**

In this study, we recruited 36 patients, including two neonates, 17 infants and 17 children. Seven cases underwent an MRI examination, while the others had a CT scan. Polygyria and pachygyria malformation were the most common type of congenital neurological malformations (7 cases, 31.8%), followed by cystic changes of the cerebral parenchyma (3 cases, 13.6%). Cerebral atrophy was the most common type of secondary CNS abnormality (8 cases, 57.1%), followed by communicative hydrocephalus (3 cases, 21.4%). Five patients in the congenital group and 4 patients in the secondary group had complex malformations. In the current study group, there were 8 deaths, 12 cases with neurological sequelae, 1 case with normal development, and 15 cases lost to follow-up. There were no significant differences between the primary and secondary CNS groups in terms of the outcome for both the infants and children groups.

**Conclusions:**

CNS malformations in the Tibetan Plateau are associated with high mortality and morbidity rates. Better utilization of imaging modalities could help design tailored treatments as early as possible.

## 1 INTRODUCTION

Neonatal nervous system malformations, which are serious congenital disabilities, have a high mortality rate and disability rate, making them a significant factor in reducing the quality of the newborn population. Tibetan areas are situated on the plateau (with an average altitude of over 3600 meters) and are sparsely populated; therefore, implementing early and standardized prenatal screening is more difficult than in mainland of China. Since 2010, the government of the Autonomous Region has established the primary working group for the prevention of birth defects, and with the support of mainland experts, prenatal ultrasound examinations have been gradually standardized, which has played a vital role in reducing the incidence of birth defects and improving the quality of the population [1]. With the gradual popularity of prenatal screening ultrasound, it is crucial to reduce the incidence of postpartum birth defects [2]. Due to delayed birth examinations or due to the lack of ultrasound diagnosis level, a considerable number of children are still born with defects. According to the data from 3128 cases in Lhasa in 2018, the neonatal birth defect was approximately 10.2% [3], indicating that the rate of neonatal birth defects in Tibetan areas is still high. The incidence of neurological malformations is gradually decreasing due to the implementation of corresponding prevention measures such as folic acid supplementation. CT or MRI methods are still crucial for evaluation after birth, as it is easier to show the anatomical details of the intracranial conditions and other accompanying neurological malformations [4]. Currently, there are relatively few imaging assessments for postnatal neurological malformations in Tibetan areas. This paper retrospectively analyzed the imaging data from our hospital over the past five years. The purpose of this study was to improve further the understanding of the types of neonatal neurological abnormalities and their corresponding imaging manifestations in very high plateau areas.

## 2 MATERIALS AND METHODS

### 2.1 Clinical Data

This study was reviewed and approved by our hospital’s ethics committee (No.2023RKZRMYY10M002). A retrospective analysis was conducted on the imaging data of patients admitted to both the neonatal and neurology departments of our hospital over the past five years, suspected of having intracranial nervous system developmental abnormalities. The enrollment criteria included an age range from newborn to 10 years old, imaging cases of suspected neurological abnormalities, and complete imaging data (CT or MRI). The exclusion criteria included patients whose images were not examined at our hospital or, patients with image artifacts that significantly affected the diagnosis, and patients exceeding the age limit.

### 2.2 CT and MRI Scan Parameters

The CT machine used was a 64-layer uCT760 (United Imaging Co.Ltd). The head CT scan parameters were as follows: scanning voltage Kv = 120, scanning current = 184mA, scanning time = 12.3s, CTDlv = 54.99mGy, CTDlp10 = 57.69mGy, scanning field of volume (FOV) = 500mm, thickness = 1.5mm, and image resolution = 512 × 512. The MRI machine used was a 3.0T uMR770 (United Imaging Co.Ltd), with a head-first scanning direction and a head coil. The scanning parameters were as follows: T1WI: TR / TE = 2024/9.24ms, thickness = 5mm, FOV = 320 × 320, resolution = 504 × 504 × 21, bandwidth = 180Hz. T2WI: TR / TE = 4619/94.4ms, flip angle = 145°, FOV = 576 × 471, resolution = 432 × 375 × 21, and bandwidth = 200Hz. The diffusion weighted imaging techniques (echo planar technique) were as follows: TR / TE = 2432/127.6ms, *b* = 1000, thickness = 5mm, resolution = 192 × 192 × 21, FOV = 243 × 216, bandwidth = 1820 Hz. The routine scanning directions included transverse, sagittal, and coronal positions.

### 2.3 Image Interpretations

The enrolled patients were admitted by two highly experienced physicians. Image diagnosis is interpreted in the following order: if the image is abnormal, it should include the following information: 1. supra infratentorial abnormalities, or both; 2. gyrus abnormalities (absence of gyrus, giant gyrus, small gyrus, or gyrus dysplasia); 3. the ventricular enlargement, subdural space effusion (blood); 4. encephalomalacia focus (perforation anomaly); 5. active cerebral hemorrhage; 6. the parenchyma volume reduction (atrophy), hydrocephalus; 7. the corresponding skull and spinal cord lesions. The included cases were divided into two groups: primary and secondary (cerebral atrophy, cerebral ventricular puncture, hydrocephalus, and subdural fluid) CNS development abnormalities. All conclusions were determined based on a consistent diagnosis after a discussion between two physicians. Follow-up for the enrolled cases was primarily conducted *via* telephone and included the following: 1) final outcome, 2) current neurological symptoms, 3) medication, and 4) family history, among others.

### 2.4 Statistical Analysis

Measurement data were expressed as mean ± standard deviation. A t-test was used to assess differences between quantitative data, and the non-parametric rank sum test was applied to qualitative data. All data were statistically analyzed using SPSS19.0 software with the test criterion α =0.05.

## 3 RESULTS

### 3.1 Clinical Characteristics

**Fig. (1):**
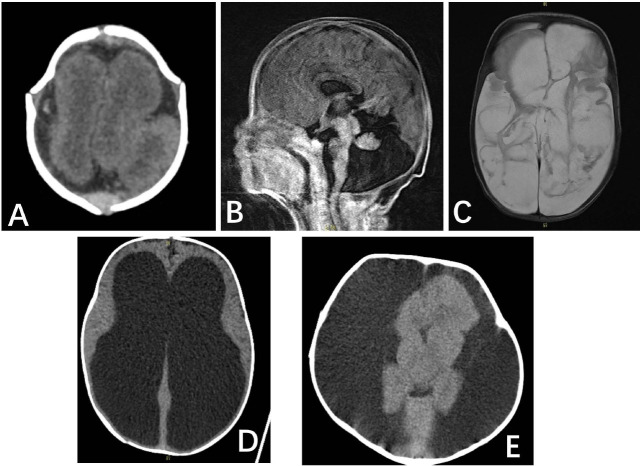
**A** One-month-old newborn. CT showed the lissencephaly malformation; **B**, one-month-old newborn, MRI showed Dandy-walker malformation with corpus callosum deficiency, he died three months after birth; **C**, two-month-old newborn, T2WI showed cystic changes in the bilateral cerebral hemisphere; **D**, two-months-old newborn, CT indicated severe hydrocephalus with thinning of cerebral cortex who died 1 month after birth; **E**, 10-months-old infant, CT showed most of hemisphere was absent with massive effusion under the skull who died one year later.

A total of 36 clinical cases were finally enrolled in this study, including 19 cases under 1 year of age, 7 cases aged between 1 and 3 years, and 10 cases over 3 years of age. Among them, 22 cases were congenital malformations, and 14 were secondary developmental abnormalities. Seven cases underwent MRI after birth, and the others had CT examinations. The mean age of newborns, infants, and pediatric groups was 7.16 ± 4.14 months, 25.3 ± 8.18 months, and 64.8 ± 18.1 months, respectively. Clinical symptoms mainly included epilepsy, muscle weakness, speech and mental impairment, and abnormal head enlargement. The follow-up periods ranged from 3 to 42 months, with a median time of approximately 26 months. In the neonatal and infant group, 8 patients died (Fig. **1**),9 patients were lost to follow-up, and 3 patients had perceptible neurological symptoms. In the children group, 1 patient had developmental delay, 3 had movement disorders, 2 had speech disorders, and 6 were lost to follow-up. All clinical signs and their corresponding follow-up results are summarized in Table **1**.

### 3.2 Imaging Findings

**Fig. (2):**
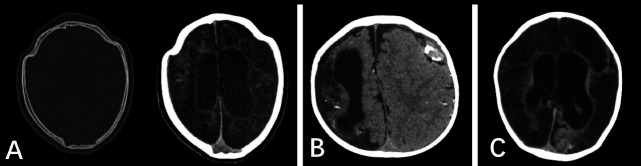
**A** Two-year-old child CT showed an irregular skull contour, suggesting craniosynostosis. Soft tissue window (right) showed extensive low-density changes in the cerebral parenchyma; **B**, a two-year-old child CT presented with dysplasia of the right cerebral hemisphere with partial penetrating malformation where some scattered high densities were observed. Under the left skull, there was a slightly circular high-density calcification, suggesting old hematoma changes; **C**, a three-year-old child with brain parenchyma, showed extensive low-density changes.

In this study, 22 cases had primary neurological malformations,14 cases had secondary CNS development abnormalities, and all imaging findings were supratentorial abnormal changes. Polygyria and pachygyria malformations were the most common type of congenital neurological malformations (7 cases, 31.8%), followed by cystic changes of the cerebral parenchyma (3 cases, 13.6%). Cerebral atrophy was the most common type of secondary CNS abnormality group (8 cases, 57.1%), followed by communicative hydrocephalus (3 cases, 21.4%). In the neonatal group, primary CNS malformations mainly included the absence of gyrus, Dandy-Walker malformation, polygyria, and partial gyri malformation. In the infant group, the congenital neurological malformations mainly included craniosynostosis (Fig. **2**), cerebral cystic parenchyma, and polygyrus malformation. In the pediatric group, there mainly were abnormal gyri development in the primary malformation group (Fig. **S1**). Five patients in the congenital group and four patients in the secondary group had complex malformations (subdural effusion, ventriculomegaly, hydrocephalus, calcification, *etc*.). The detailed occurrence of neurological malformations by age level are summarized in Tables **2** and **3**. Eight cases (22.2%) died during the follow-up, and these were in both the neonatal and infant groups. 12 (33.3%) had neurological sequelae; 1 had normal development; 15 (41.7%) were lost to follow-up. There were no significant differences in the outcome between the congenital and secondary neurological malformation groups in the infant group (Table **S1**).

## 4 DISCUSSION

In the current study, gyrus malformation (31.8%) and brain atrophy (57.1%) were the most common etiologies for the primary and secondary CNS malformation group, respectively. In the neonatal group, the CNS malformations were complicated, and the prognosis was poor. Both CT and MRI effectively depict the CNS anomalies in this study and provide valuable information for subsequent treatment and evaluation.

Theoretically, owing to the specific climate environment in the high-altitude area, physical, structural, and functional alterations in native Tibetans can be observed. For example, erythema is more commonly found in Tibetans and dwellers who have lived for a long time in high-altitude plateaus [5-8]. However, how these changes could affect the fetus or infants is still not clear. Besides, congenital infections or teratogenic factors also may affect the potential CNS of the children's occurrence. It was reported that the rate of pathogenic findings on exome in fetus screening ranged from 3% to 55% [9]. Some CNS malformations are also associated with different metabolic diseases [10, 11]. In the Tibetan plateau, medical equipment and awareness of prenatal screening remain relatively inadequate among the Zang people despite the establishment of related birth defect prevention programs in the autonomous region. The gradual popularity of prenatal ultrasound screening [12], to some extent, has reduced the rate of serious birth defects, but cultural tradition, in addition to hygiene and traffic conditions, are still the concerning factors for the incidence of severe neonatal CNS malformations [13]. Intrauterine infections also are responsible for the increasing risk of fetal CNS malformations [10]. Further, antiepileptic drugs and abuse of alcohol, which is a common scenario in the Tibetan plateau, may also promote the rate of CNS malformations in the fetus.

MRI is mainly used for the evaluation of neonatal brain atrophy, brain injury, and intracranial infections. The etiology of neonatal cerebral atrophy is complex, and it is also the most common type of CNS development abnormality in the secondary group of this studied group. Several studies have reported that the sensitivity of MRI diagnosis is significantly better than that of ultrasound examination for non-hemorrhagic brain injury. MR spectroscopy imaging (MR spectrum, MRS) or magnetic sensitivity imaging (susceptibility-weighted imaging, SWI) is used to detect early metabolic changes, especially in the assessment of cerebral hemorrhage and circulatory disorders [14-16].

Abnormal development of the gyrus begins in the cerebral cortex during fetal development, including abnormal cell proliferation and differentiation, neuronal migration disorders, and abnormal cortical tissue links after migration [15], which is also the most common primary CNS malformation in the infant group. Fetal MRI is crucial for the early judgment of abnormal cortical development, and MRI can generally be performed in suspected cases before 24 gestational weeks [16]. A limitation is a requirement for professional diagnostic physicians to interpret the images, as some normal anatomical variations may also be observed, requiring necessary prenatal consultation. The clinical manifestations of abnormal gyrus development, such as early feeding disorders, cranial enlargement, seizures, and so on have been reported [17, 18]. After growing up, they are also accompanied by movement disorders, aphasia, deafness, and other related symptoms causing the corresponding social communicative difficulties. The severity of the symptoms is also closely related to the prognosis. In the current data, the infant and neonatal groups had a poor prognosis, including severe neurological malformations and cortical development delay due to severe hydrocephalus. During the fetal period, intracranial hemorrhage is not uncommon and can result in hydrocephalus. Whether the rate of intracranial bleeding in high-altitude levels increases is not fully investigated. Although there were no deaths in the children group, they were mostly accompanied by physical or intellectual and speech development disorders. Only one patient with mild brain atrophy had no abnormal symptoms at the current time point of follow-up. Cystic change of the cerebral parenchyma was the most common image finding in the secondary CNS group in the present study. The true mechanism is unclear and may be related to previous active bleeding or clot formation in the intracranial vascular system.

The main limitations of this paper are as follows: first, it is a retrospective analysis of neonatal and pediatric congenital disability patients in the Tibetan region. The most recent population data shows fewer than 800,000 residents. The city oversees 17 counties, many of which are remote and scattered. The high-level natural conditions limit the data sample in this study. Further research covering more areas of Tibet would provide more convincing results. Second, we mainly studied the manifestations and characteristics of CT and MRI imaging, while ultrasound is still the most common method of prenatal screening [18]. Furthermore, due to the limited conditions (incomplete ultrasound report and inconsistent ultrasound reports), we did not compare the results of radiographic images with the results of ultrasound, which is also one of the deficiencies of this study. Finally, although we did posttreatment follow-up, the relatively short follow-up period and high rate of follow-up loss limited further evaluation of potential neurological sequelae.

## CONCLUSION

In conclusion, CNS developmental malformations in newborns and children in Tibetan areas are complex and associated with high mortality and disability rates. The effective use of imaging methods can aid in creating an appropriate treatment plan at an early stage.

## Figures and Tables

**Table 1:** Clinical indications and the final prognosis for all the included cases in this single respective study.

Age Group	Numbers	Clinical Indications	Outcomes
<= one year	19	seizure (N = 4), head enlargement (N = 4), vomit (N = 1), neonatal asphyxia (N = 1), muscle weakness strength (N = 2), unspecific indications (N = 7)	death (8), follow-up lost (6), visual dysfunction (1), dyskinesia and aphasia (3), no severe neurological complications (1)
Between 1year to 3 years	7	aphasia (N = 1), muscle weakness strength (N = 1), seizure (N = 1), intracranial hemorrhage (N = 3), unspecific indications (N = 1)	dyskinesia and aphasia (3), no neurological complications (1), follow-up lost (3),
> three years	10	dysgnosia (N = 1), muscle weakness strength (N = 1), dyskinesia (N = 3), seizure (N = 3), unspecific indications (N = 2)	developmental delay (N =1), movement dysfunctional (N = 2), speech impediment (N = 1), follow-up lost (6)

**Table 2:** Abnormal imaging findings in the included thirty-six cases underwent both CT or MRI examination in our institution.

Age Group	Numbers	Applied Modality	Imaging Signs	
-	-	-	With Primary Malformation (N = 22)	With Secondary Malformation (N = 14)
<= one year	19	MRI (N =3) CT (N = 16)	lissencephaly (N = 1), Dandy-walker and corpus callosum hypoplasia (N = 1), partial absence of gyrus (N =2), polygyria (N =1), cystic deformation of cerebral parenchyma (N = 2)	communicative hydrocephalus (N = 3), encephalatrophy (N = 7), porencephaly (N = 2)
Between 1year to 3 years	7	CT (N = 7)	craniosynostosis (N = 2), porencephaly (N = 1), polygyria (N = 2), cystic deformation of cerebral parenchyma (N = 3)	-
> three years	10	MRI (N = 4) CT (N = 6)	partial absence of gyrus (N =1), polygyria or pachygyria (N = 4), dysplasia of the gyrus (N = 2), porencephaly (N = 1)	ventriculomegaly (N =1), encephalatrophy (N = 1)

**Table 3:** Summarized abnormal imaging findings in the twenty-one cases with the follow-up conditions.

Age Group	Numbers	Applied Modality	Imaging Signs	
-	-	-	With Primary Malformation (N = 4)	With Secondary Malformation (N = 14)
<= one year	13	MRI (N =2) CT (N = 11)	lissencephaly (N = 1), Dandy-walker and corpus callosum hypoplasia (N = 1), porencephaly (N = 1), polygyria (N =1)	hydrocephalus (N = 2), encephalatrophy (N = 7), porencephaly (N = 2), cerebral atrophy (N = 6), subdural effusion (N = 3), cortical dysplasia (N = 2)
-	-	Outcome	No perceptible complications = 2, Death = 2,	No perceptible complications = 2, Death = 4, Neurological complications = 3 (one with strabismus, two with aphasia)
Between 1year to 3 years	4	CT (N = 4)	craniosynostosis (N = 2), porencephaly (N = 1), polygyria (N = 2), subdural effusion (N = 1), cerebral atrophy (N = 1), cystic deformation of cerebral parenchyma (N = 1)	-
-	-	Outcome	no perceptible complications = 1, Neurological complications = 3(movement and speech disorder)	-
> three years	4	MRI (N = 1) CT (N = 3)	partial absence of gyrus (N =1), polygyria or pachygyria (N = 1), porencephaly (N = 1)	porencephaly and ventriculomegaly (N =1),
-	-	Outcome	development delay = 1, neurological complications = 2 (movement and speech disorder)	neurological complications = 1 (movement disorder)

## Data Availability

The data and supportive information are available within the article.

## LIST OF ABBREVIATIONS

CNS: Central nervous system
MRI: Magnetic resonance imaging
CT: Computed tomography
FOV: Field of volume

## STANDARDS OF REPORTING

STROBE guidelines were followed.
