# Supplementary material for: Intracranial Structural Malformations in Children in Tibet: CT and MRI Findings in a Single Tertiary Center
Source: Curr Med Imaging. 2025 Jan 2;21:e15734056321642. doi: 10.2174/0115734056321642241213103658 (PMC12813543; doi:10.2174/0115734056321642241213103658)
Supplement: Supplementary file 1 [file CMIM-21-E15734056321642_SD1.pdf]

1573-4056/XX

Send Orders for Reprints to reprints@benthamscience.net

BENTHAM SCIENCE

CrossMark

Current Medical Imaging

Content list available at: https://benthamscience.com/journals/cmir

Supplementary Material

Intracranial Structural Malformations in Children in Tibet: CT and MRI Findings in a Single Tertiary Center

Xuan Yin[1,#], Dawa Ciren[2,#], Ciren Guojie[2], Guofu Zhang[1], Jimei Wang[3] and He Zhang[1,2,*]

[1]*Department of Radiology, Obstetrics and Gynecology Hospital, Fudan University, Shanghai, P.R.China*
[2]*Department of Radiology, Shigatse People's Hospital, Shigatse, Xianzang Autonomous Region, P.R.China*
[3]*Department of Neonatology, Obstetrics and Gynecology Hospital, Fudan University, Shanghai, P.R.China*

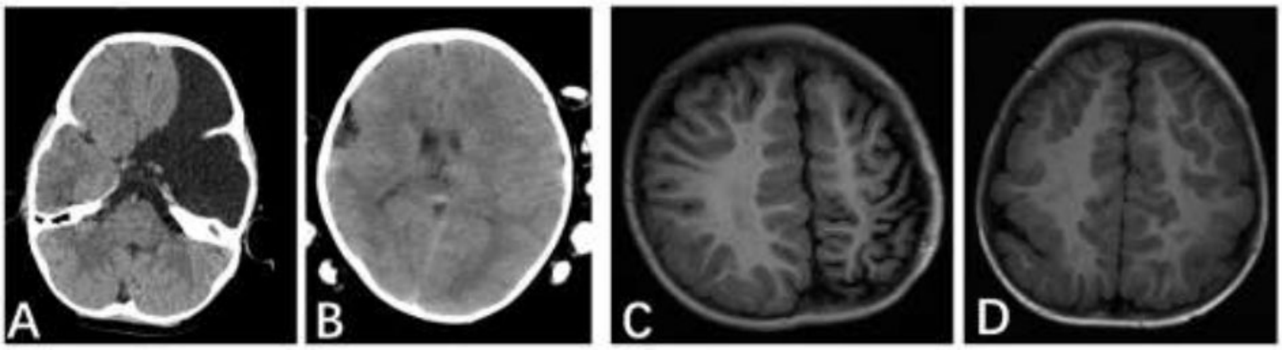

Fig. (S1). A, 48-months-child, CT indicated the absence of the left frontal lobe. Follow-up results detect his unclear speech with normal movement function; **B**, 60-months-child, CT showed the giant gyrus in the right hemisphere and the polygyria in the left hemisphere; **C**, 48-months-child, MRI disclosed the dysplasia of both the front and parietal lobe in the left hemisphere; **D**, 48-months-child, MRI indicated bilateral polygyria in both frontal and parietal lobe.

Table S1. The outcomes comparison between the two different malformation groups across the age level.

**Age Group**	**Numbers**	**With Primary Malformation (N = 22)**	**With Secondary Malformation (N = 14)**	**P = 0.038**
<= 1 year	19	-	-	P = 0.332
normal	-	-	1	-
death	-	3	5	-
neurological sequelae	-	1	3	-
loss	-	3	3	-
Between 1 year to 3 years	7	-	-	NA
normal	-	-	-	-
death	-	-	-	-
neurological sequelae	-	4	-	-
loss	-	3	-	-
	-	-	-	-
> 3 years	10	-	-	0.759

DOI: 10.2174/0115734056321642241213103658, XXXX, *XX*, e15734056321642

Age Group	Numbers	With Primary Malformation (N = 22)	With Secondary Malformation (N = 14)	P = 0.038
normal	-	-	-	-
death	-	-	-	-
neurological sequelae	-	3	1	-
loss	-	5	1	-

© 2024 The Author(s). Published by Bentham Science Publisher.

This is an open access article distributed under the terms of the Creative Commons Attribution 4.0 International Public License (CC-BY 4.0), a copy of which is available at: https://creativecommons.org/licenses/by/4.0/legalcode. This license permits unrestricted use, distribution, and reproduction in any medium, provided the original author and source are credited.

DISCLAIMER: The above article has been published, as is, ahead-of-print, to provide early visibility but is not the final version. Major publication processes like copyediting, proofing, typesetting and further review are still to be done and may lead to changes in the final published version, if it is eventually published. All legal disclaimers that apply to the final published article also apply to this ahead-of-print version.
